# Soil Inorganic Carbon Sequestration Following Afforestation Is Probably Induced by Pedogenic Carbonate Formation in Northwest China

**DOI:** 10.3389/fpls.2017.01282

**Published:** 2017-07-19

**Authors:** Yang Gao, Jing Tian, Yue Pang, Jiabin Liu

**Affiliations:** ^1^State Key Laboratory of Soil Erosion and Dryland Farming on the Loess Plateau, Northwest A&F University Yangling, China; ^2^College of Forestry, Northwest A&F University Yangling, China; ^3^College of Natural Resources and Environment, Northwest A&F University Yangling, China

**Keywords:** afforestation, degraded semiarid regions, pedogenic inorganic carbon, soil inorganic carbon, stable carbon isotope

## Abstract

In arid and semiarid areas, the effects of afforestation on soil organic carbon (SOC) have received considerable attention. In these areas, in fact, soil inorganic carbon (SIC), rather than SOC, is the dominant form of carbon, with a reservoir approximately 2–10 times larger than that of SOC. A subtle fluctuation of SIC pool can strongly alter the regional carbon budget. However, few studies have focused on the variations in SIC, or have used stable soil carbon isotopes to analyze the reason for SIC variations following afforestation in degraded semiarid lands. In the Mu Us Desert, northwest China, we selected a shifting sand land (SL) and three nearby forestlands (*Populus alba*) with ages of 8 (P-8), 20 (P-20) and 30 (P-30) years, and measured SIC, SOC, soil organic and inorganic δ^13^C values (δ^13^C-SOC and δ^13^C-SIC) and other soil properties. The results showed that SIC stock at 0–100 cm in SL was 34.2 Mg ha^-1^, and it increased significantly to 42.5, 49.2, and 68.3 Mg ha^-1^ in P-8, P-20, and P-30 lands, respectively. Both δ^13^C-SIC and δ^13^C-SOC within the 0–100 cm soil layer in the three forestlands were more negative than those in SL, and gradually decreased with plantation age. Afforestation elevated soil fine particles only at a depth of 0–40 cm. The entire dataset (260 soil samples) exhibited a negative correlation between δ^13^C-SIC and SIC content (*R*^2^ = 0.71, *P* < 0.01), whereas it showed positive correlation between SOC content and SIC content (*R*^2^ = 0.52, *P* < 0.01) and between δ^13^C-SOC and δ^13^C-SIC (*R*^2^ = 0.63, *P* < 0.01). However, no correlation was observed between SIC content and soil fine particles. The results indicated that afforestation on shifting SL has a high potential to sequester SIC in degraded semiarid regions. The contribution of soil fine particle deposition by canopy to SIC sequestration is limited. The SIC sequestration following afforestation is very probably caused by pedogenic carbonate formation, which is closely related to SOC accumulation. Our findings suggest that SIC plays an important role in the carbon cycle in semiarid areas and that overlooking this carbon pool may substantially lead to underestimating carbon sequestration capacity following vegetation rehabilitation.

## Introduction

Arid and semiarid areas cover approximately 41% of the Earth’s land surface (Reynolds et al., 2007; Delgado-Baquerizo et al., 2013). In these areas, desertification is an extremely challenging environmental problem leading to serious land degradation and enormous losses of soil carbon (Lal, 2009; Li et al., 2015). However, if appropriate restoration measures can be successfully implemented on degraded lands, it is possible to effectively curb land degradation and substantially improve the soil properties in these lands (Lal, 2004; Huang et al., 2012). Afforestation is an important restoration measure for degraded lands and is generally considered to have great potential to combat desertification, protect soils and alter the soil carbon pool (Lal, 2010). The soil carbon pool comprises the soil organic carbon (SOC) and soil inorganic carbon (SIC) pools (Zhang et al., 2015). Because of its potentially rapid response to afforestation, the SOC pool has received considerable attention and has been extensively investigated (Jackson et al., 2002; Deng et al., 2014). In contrast to the great progress made in understanding the dynamics of the SOC pool, the effects of afforestation on the SIC pool have received relatively less consideration (Wang et al., 2010; Meyer et al., 2014). In fact, SIC, rather than SOC, is the dominant form of carbon in arid and semiarid areas (Mielnick et al., 2005; Mi et al., 2008), with a reservoir approximately 2–10 times larger than that of SOC (Schlesinger, 1982; Tan et al., 2014). Due to the large reservoir of SIC, a subtle fluctuation in the SIC pool will strongly alter the carbon budget in arid and semiarid areas (Landi et al., 2003; Jin et al., 2014). It is therefore important to have a thorough understanding of the dynamics of SIC pool following afforestation in these regions.

Changes in SIC following afforestation in arid and semiarid areas exhibit contrasting trends, some of which are in direct opposition. For instance, in the Horqin Sandy Land and Badain Jaran Desert, China, planting Mongolian pine and poplar significantly stimulated the accumulation of SIC (Su et al., 2010; Li Y.Q. et al., 2013). In contrast, in the Columbia Plateau of Oregon, United States, poplar afforestation was found to reduce the SIC stock (Sartori et al., 2007). Another study in the Loess Plateau of China reported that afforestation simply redistributed SIC along the soil profile without affecting its total quantity (Chang et al., 2012). These results indicate that the effects of afforestation on SIC stock need to be further examined in arid and semiarid areas.

Importantly, uncertainty nonetheless remains as to why SIC showed variation following afforestation. There are several geological methods (such as scanning electron microscopes) for studying SIC variations (Zamanian et al., 2016). Among these, stable soil carbon isotopes (^13^C) have been demonstrated to be an applicable and crucial indicator revealing the reason for SIC variations following land use changes (Cerling et al., 1989; Stevenson et al., 2005). The SIC pool consists of lithogenic inorganic carbon (LIC) and pedogenic inorganic carbon (PIC) pools, and these two subpools have different δ^13^C values (Jobbágy and Jackson, 2003; Chang et al., 2012; Tan et al., 2014). The LIC subpool is inherited from the parent material and generally has high δ^13^C values (close to zero), whereas the PIC subpool is generated from the precipitation of carbonate ions and generally shows low δ^13^C values (negative) (Wang et al., 2016; Zamanian et al., 2016). The dynamics of the SIC pool following land use changes are dominated by the LIC and PIC subpools. Various processes in SIC variations, including the mixing of LIC with PIC and the reaction of soil carbonate with biogenic CO_2_, can be sensitively and precisely reflected in δ^13^C values (Stevenson et al., 2005; Monger et al., 2015). The use of stable soil carbon isotopes method, in which the soil inorganic δ^13^C value (δ^13^C-SIC) and the soil organic δ^13^C value (δ^13^C-SOC) are measured, has been found to be an ideal approach to studying the inherent mechanisms of SIC dissolution, sequestration and transformation following land use changes (Stevenson et al., 2005; Rao et al., 2006; Li G.J. et al., 2013; Wang J.P. et al., 2015). In arid croplands, determining the changes in δ^13^C-SIC and δ^13^C-SOC following straw organic amendments, revealed that such amendments enhanced PIC formation and led to SIC accumulation (Wang et al., 2014; Wang X.J. et al., 2015). In semiarid restored grassland, a decrease in δ^13^C-SIC indicated that soil carbonate exchanged with biogenic CO_2_, resulting in lower SIC stock in grassland than in farmland (Liu et al., 2014). Despite the value provided by the existing carbon isotope methods, they have not been extensively utilized to explore the reason for SIC variations after afforestation in degraded semiarid lands, particularly for afforestation on shifting sand land (SL).

Sand land, which is widely distributed in northwest China, is characterized by extreme deterioration of the plant and soil environment. Afforestation and shrub-planting are commonly suggested as options to combat desertification (Zhang K. et al., 2010; Zhang Y. et al., 2013). Previous studies have conclusively demonstrated that afforestation on SL significantly promotes SOC storage (Liu et al., 2013; Li et al., 2016). However, few studies have focused on the variations in SIC, or have used stable soil carbon isotopes to analyze the mechanisms underlying SIC variations following afforestation on SL. The use of the related field data along a chronosequence of afforestation, which could more precisely and reliably determine the dynamics of SIC, has rarely been reported. The changes in soil carbon along a chronosequence of afforestation are often studied by comparing the different-aged forestlands within a designated area (space-for-time substitution approach) (Farley et al., 2004; Qiu et al., 2015), as the historical data in a same forestland since the beginning of afforestation cannot be obtained at present. In view of the above deficiencies, we selected an SL and three nearby forestlands (*Populus alba*) with ages of 8 (P-8), 20 (P-20), and 30 (P-30) years within 2 km^2^ in the Mu Us Desert, northwest China. We measured SIC, SOC, δ^13^C-SOC and δ^13^C-SIC in both the SL and the three different-aged forestlands at depth of 100 cm. The objectives of this research were (1) to examine the changes in SIC along a chronosequence of afforestation and (2) to explore the reasons for SIC variations following afforestation using the carbon isotope method.

## Materials and Methods

### Study Site Description

**FIGURE 1:**
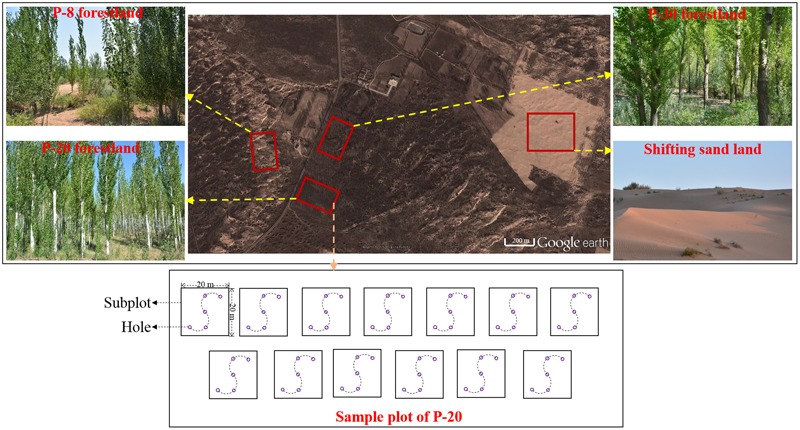
Distribution of the four sample plots in the study area and the relationships between sample plot, subplot and hole. The satellite image was obtained from Google Earth and was taken in January 2013. Other pictures were taken by YG in September 2015.

The study site is located at the Station of Chunlan Bai Desertification Control, Yanchi County, Ningxia Province, China (107°27′ E, 37°54′ N), on the southwestern edge of the Mu Us Desert. The region has a typical temperate continental monsoon climate with an elevation of 1308 m. The mean annual precipitation is 275 mm, with 73% occurring in summer and autumn. The mean annual temperature is 7°C. The average relative humidity is 51% and the frost-free period lasts for 128 days. According to the US Soil Taxonomy system, the soil type is quartisamment (Gao et al., 2014), with a pH range of 8.0 to 9.0. In the 1980s, the landscape of the research area was dominated by SL, which comprised many connected active sand dunes devoid of any vegetation. At that time, the groundwater level was high enough (2 m) to supply water for tree growth. Afforestation with poplar (*Populus alba*) on SL was successively performed by Chunlan Bai and her family to restrict sand movement and to protect their homeland. At present, forestlands with different plantation ages have been established at the study site. Additionally, areas of SL at some distance from human habitation have not been managed, and have remained active. Previous studies have confirmed that the soil properties in the SL do not vary over a prolonged period of time (Su and Zhao, 2003; Su et al., 2010), suggesting that the soil properties prior to the start of the experiments can be represented by those in the SL at the time of the study. Therefore, the present-day SL can be used as a control for investigating the changes in SIC and soil stable carbon isotopes following afforestation. In this study, we used different-aged forestlands to explore the dynamics of SIC along a chronosequence of afforestation, because there had been no related study in this region and there was a lack of historical data. Within the scope of the 2 km × 1 km in the study site, we selected an SL and its nearby three different-aged forestlands as the four treatments: (1) the SL (control), (2) an 8-year-old poplar land, (3) a 20-year-old poplar land, and (4) a 30-year-old poplar land. For each treatment, we selected one sample plot. The distribution of the four sample plots within the study site is illustrated in **Figure 1**, and information on the four sample plots is presented in **Table 1**.

**Table 1:** Characteristics of the four sample plots (mean ± standard deviation; *n* = 13).

Plots	Unit	SL	P-8	P-20	P-30
Sample plot area	ha	4	3	3	3
Plant species		–	*Populus alba*	*Populus alba*	*Populus alba*
Density	trees ha^-1^	0	585	543	502
Height	m	0	6.4 ± 1.1	12.8 ± 2.2	15.5 ± 2.7
Diameter at breast height	cm	0	6.2 ± 0.8	15.8 ± 1.9	22.6 ± 2.7
Coverage	%	0	28.9	35.7	40.2
Soil electrical conductivity	dS m^-1^	4.52 ± 0.37	4.73 ± 0.26	4.68 ± 0.24	4.86 ± 0.38
Ca^2+^ in soil	cmol kg^-1^	4.79 ± 0.24	4.68 ± 0.35	4.92 ± 0.21	5.01 ± 0.18
Mg^2+^ in soil	cmol kg^-1^	0.33 ± 0.08	0.41 ± 0.04	0.39 ± 0.05	0.48 ± 0.05
Soil total porosity	%	40.3 ± 0.8	43.5 ± 0.7	44.6 ± 0.5	45.7 ± 0.7

### Soil Sampling and Analyses

Thirteen 20 m × 20 m subplots were randomly selected within each sample plot for soil sampling. In each subplot, five holes (100 cm in depth) along an S-shaped curve were drilled using a soil auger (10 cm in diameter) after removing litter (the relationships between sample plot, subplot and hole are shown in **Figure 1**). The soil samples were obtained at a depth interval of 20 cm from 0 to 100 cm. In each subplot, five soil samples obtained from five holes at the same layer were mixed into a composite sample (approximately 500 g), and five composite samples were achieved at a depth interval of 20 cm from 0 to 100 cm within each subplot. Sixty-five composite samples from the 13 subplots within each sample plot were obtained. After the samples were air-dried, roots were removed from all the 260 composite samples from the four sample plots. For each air-dried composite sample, approximately 50 g soil was taken and retained for measuring particle size distribution using a particle size analyzer (Malven Laser Mastersizer 2000, England). The remaining air-dried composite samples were fully ground in an agate mortar and passed through a 0.1 mm sieve for SIC content, SOC content and soil δ^13^C analyses.

After obtaining the 260 composite samples, a soil profile at 0–100 cm was excavated within each subplot. A metal corer (100 cm^3^ in volume) was driven into the soil at a depth interval of 20 cm from 0 to 100 cm, and then soil samples were oven dried at 115°C for 24 h and weighed to determine bulk density. From the excavated soil profile in each subplot, additional soil samples were obtained at a depth interval of 20 cm from 0 to 100 cm for measuring Soil pH, using a 2.5:1 ratio of deionized water/soil mass. SOC content was determined using the dichromate oxidation procedure described by Walkley and Black (1934). SIC content was determined using the pressure calcimeter method (Wang et al., 2012). The stocks of SIC were calculated as follows:

(1) M=0.1×D×B×Z×((100-G)/100)

where M is soil carbon stock per unit area (Mg ha^-1^); D is soil depth (cm); B is bulk density (g cm^-3^); Z is carbon content (g kg^-1^) and G is the relative amount of gravel (%). The gravel content was 0 because there was no gravel in the soil.

The detailed methods for determining δ^13^C-SOC and δ^13^C-SIC have been described previously by Jin et al. (2014). For the determination of δ^13^C-SOC, 5 g of ground and sieved soil was steeped in 2 M HCl for 24 h to remove SIC. The treated soil was then washed with distilled water until the pH exceeded 5, and was subsequently dried at 40°C. From each dried soil sample, approximately 30 mg soil was packed in a tin cup and analyzed with an elemental analyzer (Flash EA 1112, Thermo Fisher Scientific, Inc.) and an isotope ratio mass spectrometer (IRMS) (Finnigan MAT Delta plus XP, Thermo Fisher Scientific, Inc.). The contents of the tin cup were combusted at 1000°C in the EA, and then the SOC of the sample in the tin cup was converted to CO_2_. The CO_2_ from the EA was ionized and its δ^13^C value was measured by IRMS. The working standards used for determining δ^13^C-SOC were Protein (Elemental Analyses, Inc., Beijing, China, -26.98‰) and NBS-19 (National Institute of Standards and Technology, Gaithersburg, MD, United States; +1.95‰).

To determine ^13^C-SIC, approximately 100 mg sieved soil was reacted with 5 mL 100% H_3_PO_4_ for 2 h at 75°C in a 12 mL sealed vessel of Gas Bench II (Thermo Fisher Scientific, Inc.) to generate CO_2_, and the generated CO_2_ was measured by IRMS (Finnigan MAT Delta plus XP, Thermo Fisher Scientific, Inc.). The working standards used for determining δ^13^C-SIC were NBS-18 (National Institute of Standards and Technology, Gaithersburg, MD, United States; -5.01‰) and NBS-19.

The stable isotope compositions of the SOC and SIC, expressed in delta (δ) notation, were both calculated as follows (Coplen, 2011):

(2) $$\delta^{\text{13}}\text{C=}\frac{{{(}^{\text{13}}{\text{C/}}^{\text{12}}\text{C})}_{\text{sample}}}{{{(}^{\text{13}}{\text{C/}}^{\text{12}}\text{C})}_{\text{standard}}}\text{-1}$$

where (^13^C/^12^C)_sample_ and (^13^C/^12^C)_standard_ are the atomic ratio of ^13^C to ^12^C in the sample and in the Vienna Pee Dee Belemnite (VPDB) standard, respectively. All samples were measured in triplicate. In the three measurements for each sample, the standard deviation of the reported δ^13^C-SOC and δ^13^C-SIC in this study was within 0.4 and 0.3‰, respectively.

### Statistical Analyses

Statistical analyses were performed using version 16.0 of the SPSS software (SPSS, Chicago, IL, United States). Two-way analysis of variance was conducted to test the effects of soil depth and plant age, as well as their interactions with soil carbon contents and soil δ^13^C values (**Table 2**). Multiple comparisons and one-way analysis of variance procedures were used to compare the differences in soil carbon contents and soil δ^13^C values between different treatments within the same depth, and between different soil depths within the same treatment. Mean comparisons were performed using the least-significant-difference test. Linear regression analyses were carried out to evaluate the relationships between various carbon variables (SOC vs. SIC, δ^13^C-SIC vs. SIC, δ^13^C-SIC vs. δ^13^C-SOC, SIC vs. silt particle, SIC vs. clay particle).

**Table 2:** Two-way ANOVA for soil carbon content, δ^13^C-SIC, and δ^13^C-SOC in for treatments and soil layers.

Soil carbon	Treatment		Layer		Treatment × Layer	
	F	P	F	P	F	P
SOC	935.7	<0.001	39.78	<0.001	7.1	<0.001
SIC	156.86	<0.001	2.27	0.062	1.82	0.046
δ^13^C-SIC	217.19	<0.001	1.81	0.128	0.31	0.986
δ^13^C-SOC	150.57	<0.001	6.45	<0.001	1.22	0.267

## Results

### Bulk Density, Soil Particle Content and pH in Shifting Sand Land and Forestlands

Afforestation was found to cause a variation in bulk density and fine particles at 0–40 cm soil layer (**Table 3**). Within this depth, the bulk densities in P-20 land and P-30 land were significantly lower than in SL, but there was no significant difference between P-8 land and SL. The silt and clay particle contents at 0–20 cm in the three forestlands were significantly higher than in SL. At the depth of 20–40 cm, the silt particle content in P-30 land was significantly greater than that in SL, but there was no significant difference between P-8 land and SL or between P-20 land and SL. The clay particle content in P-20 land was remarkably greater than in SL, but there was no significant difference between P-8 land and SL or between P-30 land and SL. Within the 40–100 cm depth layer, no differences in bulk density or fine particles were observed between the four sample plots (**Table 3**). Additionally, soil pH at 0–100 cm in P-20 land and P-30 land was considerably lower than that in SL, but there was no significant difference between P-8 land and SL within the 60–100 cm depth layer (**Table 3**).

**Table 3:** Bulk density, particle content and pH of soil in the four sample plots (*n* = 13, mean ± SD).

Soil properties	Soil depth (cm)	SL	P-8	P-20	P-30
Bulk density (g cm^-3^)	0–20	1.58 ± 0.14 a	1.51 ± 0.14 ab	1.45 ± 0.09 bc	1.43 ± 0.13 c
	20–40	1.56 ± 0.11 a	1.54 ± 0.11 ab	1.47 ± 0.15 bc	1.45 ± 0.14 c
	40–60	1.57 ± 0.13 a	1.52 ± 0.10 a	1.51 ± 0.12 a	1.49 ± 0.11 a
	60–80	1.59 ± 0.12 a	1.56 ± 0.13 a	1.55 ± 0.16 a	1.54 ± 0.09 a
	80–100	1.57 ± 0.09 a	1.58 ± 0.12 a	1.53 ± 0.13 a	1.52 ± 0.14 a

Sand (>0.05 mm, %)	0–20	91.3 ± 3.5 a	90.0 ± 3.8 a	89.7 ± 3.9 a	89.2 ± 2.9 a
	20–40	91.7 ± 2.6 a	90.7 ± 2.4 a	90.5 ± 2.8 a	90.9 ± 3.2 a
	40–60	91.9 ± 2.1 a	91.7 ± 3.2 a	91.1 ± 2.1 a	91.5 ± 3.4 a
	60–80	92.2 ± 3.2 a	91.9 ± 2.9 a	91.7 ± 3.6 a	91.6 ± 3.8 a
	80–100	92.6 ± 3.3 a	92.1 ± 2.8 a	92.6 ± 2.7 a	92.7 ± 3.9 a

Silt (0.002–0.05 mm, %)	0–20	4.8 ± 0.3 b	5.3 ± 0.4 a	5.4 ± 0.3 a	5.8 ± 0.3 a
	20–40	4.7 ± 0.2 b	5.1 ± 0.4 ab	5.0 ± 0.3 ab	5.2 ± 0.5 a
	40–60	4.9 ± 0.3 a	4.8 ± 0.3 a	5.1 ± 0.4 a	4.9 ± 0.3 a
	60–80	4.6 ± 0.2 a	4.9 ± 0.5 a	4.8 ± 0.4 a	4.7 ± 0.3 a
	80–100	4.7 ± 0.4 a	4.8 ± 0.2 a	4.6 ± 0.3 a	4.9 ± 0.4 a

Clay (<0.002 mm, %)	0–20	3.9 ± 0.4 b	4.7 ± 0.3 a	4.9 ± 0.2 a	5.0 ± 0.4 a
	20–40	3.6 ± 0.5 b	4.2 ± 0.4 ab	4.5 ± 0.5 a	3.9 ± 0.3 ab
	40–60	3.2 ± 0.6 a	3.5 ± 0.3 a	3.8 ± 0.5 a	3.6 ± 0.5 a
	60–80	3.2 ± 0.4 a	3.2 ± 0.3 a	3.5 ± 0.3 a	3.7 ± 0.4 a
	80–100	2.7 ± 0.5 a	3.1 ± 0.4 a	2.8 ± 0.4 a	2.4 ± 0.3 a

pH	0–20	8.9 ± 0.3 a	8.6 ± 0.4 ab	8.2 ± 0.3 b	8.1 ± 0.2 b
	20–40	9.0 ± 0.3 a	8.5 ± 0.3 b	8.2 ± 0.2 bc	8.0 ± 0.3 c
	40–60	8.8 ± 0.2 a	8.3 ± 0.4 ab	8.1 ± 0.1 ab	7.9 ± 0.2 b
	60–80	8.7 ± 0.1 a	8.4 ± 0.2 ab	8.2 ± 0.3 b	8.2 ± 0.1 b
	80–100	8.9 ± 0.2 a	8.5 ± 0.3 ab	8.3 ± 0.4 b	8.1 ± 0.3 b

*Within each depth, different lowercase letters denote significant differences among the treatments (*P* < 0.05).*

### SIC in Shifting Sand Land and Forestlands

**FIGURE 2:**
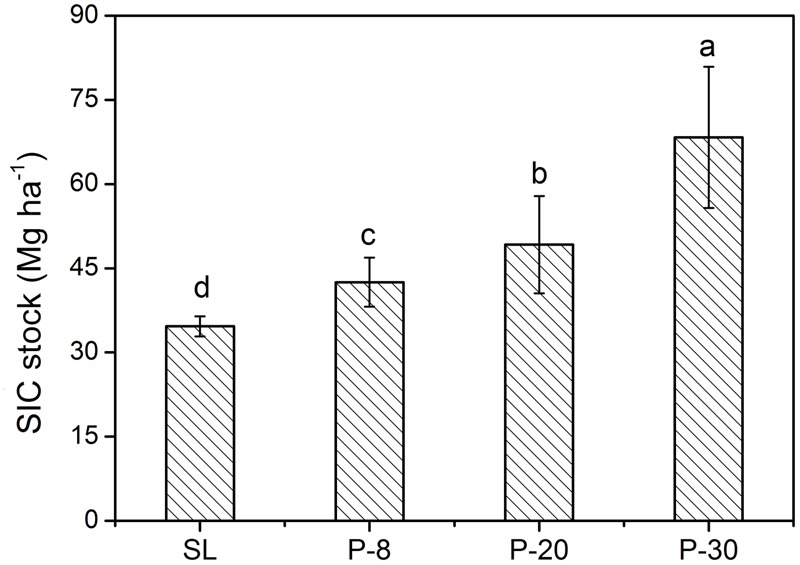
Soil inorganic carbon (SIC) stocks within 0–100 cm in shifting sand land (SL), 8-year-old poplar (P-8) land, 20-year-old poplar (P-20) land, and 30-year-old poplar (P-30) land (mean ± SD; *n* = 13). Different lowercase letters denote significant differences among the treatments (*P* < 0.05).

Soil inorganic carbon content was enhanced by afforestation. Within the 0–100 cm depth, the SIC content in each 20 cm depth interval in P-8, P-20, and P-30 lands was significantly higher than in SL (**Table 4**). Among the three forestlands, the SIC content increased with plantation age. Within the 0–40 cm layer, the SIC content in P-30 land was considerably higher than in P-20 land, but there was no significant difference between P-20 land and P-8 land. Within the 40–100 cm layer, the SIC content in P-30 land was significantly greater than that in P-20 land, which in turn was greater than that in P-8 land. Afforestation also elevated SIC stocks. The SIC stock at 0–100 cm in SL was 34.2 Mg ha^-1^, which increased to 42.5, 49.2, and 68.3 Mg ha^-1^ in P-8, P-20 and P-30 lands, respectively (**Figure 2**). The SIC contents in SL, P-8 land and P-20 land were almost evenly distributed among the five 20 cm soil intervals from 0 to 100 cm (**Table 4**). The SIC content in P-30 land at 0–20 cm was significantly higher than at 80–100 cm; however, no differences were observed among the 0–80 cm layers or among the 20–100 cm layers. In addition, the SOC content in the three forestlands was significantly higher in each soil layer than at the same depth in SL (**Table 4**).

**Table 4:** Soil carbon contents in the four sample plots (g kg^-1^; mean ± standard deviation; *n* = 13).

Soil carbon	Soil depth (cm)	SL	P-8	P-20	P-30
SIC	0–20	2.18 ± 0.22 Ac	2.77 ± 0.61 Ab	3.17 ± 0.38 Ab	5.24 ± 1.16 Aa
	20–40	2.16 ± 0.19 Ac	2.97 ± 0.27 Ab	3.25 ± 0.58 Ab	4.66 ± 0.77 ABa
	40–60	2.17 ± 0.18 Ad	2.64 ± 0.50 Ac	3.55 ± 0.70 Ab	4.59 ± 0.92 ABa
	60–80	2.19 ± 0.19 Ad	2.67 ± 0.57 Ac	3.21 ± 0.70 Ab	4.55 ± 0.86 ABa
	80–100	2.17 ± 0.20 Ad	2.74 ± 0.33 Ac	3.09 ± 0.58 Ab	3.84 ± 0.58 Ba

SOC	0–20	0.40 ± 0.03 Ad	2.00 ± 0.20 Ac	4.53 ± 0.86 Ab	6.11 ± 1.03 Aa
	20–40	0.38 ± 0.02 Ad	1.54 ± 0.21 Bc	3.88 ± 0.79 Bb	4.98 ± 0.80 Ba
	40–60	0.42 ± 0.04 Ad	1.34 ± 0.21 BCc	3.40 ± 0.59 BCb	4.69 ± 0.77 Ba
	60–80	0.38 ± 0.03 Ad	1.20 ± 0.23 CDc	3.21 ± 0.38 BCb	4.03 ± 0.40 Ca
	80–100	0.40 ± 0.03 Ad	1.03 ± 0.18 Dc	3.00 ± 0.45 Cb	3.43 ± 0.37 Ca

*Within each treatment, different uppercase letters denote significant differences among the depths (*P* < 0.05); within each depth, different lowercase letters denote significant differences among the treatments (*P* < 0.05).*

### δ^13^C-SIC and δ^13^C-SOC in Shifting Sand Land and Forestlands

In P-8, P-20, P-30 and SL lands, the δ^13^C-SIC values showed little vertical variation throughout the 0–100 cm soil layers (**Table 5**). Among the four sample plots, the δ^13^C-SIC values in SL land were the highest in all five soil layers, and δ^13^C-SIC value decreased with plantation age after afforestation. At 0–80 cm, the δ^13^C-SIC values in P-30 land were significantly lower than those in P-20 land, which in turn were lower than those in P-8 land. At 80–100 cm, δ^13^C-SIC value in P-30 land was also the lowest, but no difference was observed at this layer between P-20 land and P-8 land. The δ^13^C-SOC values within the 0–60 cm depth showed a gradual decrease with plantation age after afforestation. At 60–100 cm, the δ^13^C-SOC values were not significantly different between SL land and P-8 land, but these values in the both plots were dramatically higher than those in P-20 land and P-30 land (**Table 5**).

**Table 5:** δ^13^C-SIC and δ^13^C-SOC in the four sample plots (‰; mean ± standard deviation; *n* = 13).

	Soil depth (cm)	SL	P-8	P-20	P-30
δ^13^C-SIC	0–20	-4.08 ± 0.27 Aa	-4.72 ± 0.55 Ab	-5.59 ± 0.65 Ac	-6.72 ± 0.69 Ad
	20–40	-4.04 ± 0.23 Aa	-5.08 ± 0.63 Aa	-5.90 ± 0.47 Ab	-6.41 ± 0.68 Ac
	40–60	-4.00 ± 0.26 Aa	-4.71 ± 0.70 Ab	-5.69 ± 0.51 Ac	-6.55 ± 0.66 Ad
	60–80	-4.05 ± 0.23 Aa	-4.95 ± 0.77 Ab	-5.87 ± 0.63 Ac	-6.69 ± 0.76 Ad
	80–100	-4.06 ± 0.23 Aa	-5.17 ± 0.63 Ab	-5.74 ± 0.62 Ab	-6.58 ± 0.70 Ac

δ^13^C-SOC	0–20	-18.68 ± 1.54 Aa	-23.36 ± 2.00 Bb	-25.44 ± 1.90 Bb	-27.60 ± 2.20 Bc
	20–40	-18.82 ± 1.66 Aa	-21.46 ± 2.31 ABb	-24.41 ± 1.77 ABc	-26.75 ± 2.28 ABd
	40–60	-19.08 ± 1.89 Aa	-21.60 ± 1.98 ABb	-24.39 ± 1.52 ABc	-25.49 ± 1.47 ABc
	60–80	-19.37 ± 2.06 Aa	-20.84 ± 2.13 Aa	-23.83 ± 1.78 ABb	-25.50 ± 2.30 ABb
	80–100	-18.71 ± 1.81 Aa	-20.34 ± 2.43 Aa	-23.34 ± 1.45 Ab	-24.81 ± 2.42 Ab

*Within each treatment, different uppercase letters denote significant differences among the depths (*P* < 0.05); within each depth, different lowercase letters denote significant differences among the treatments (*P* < 0.05).*

**FIGURE 3:**
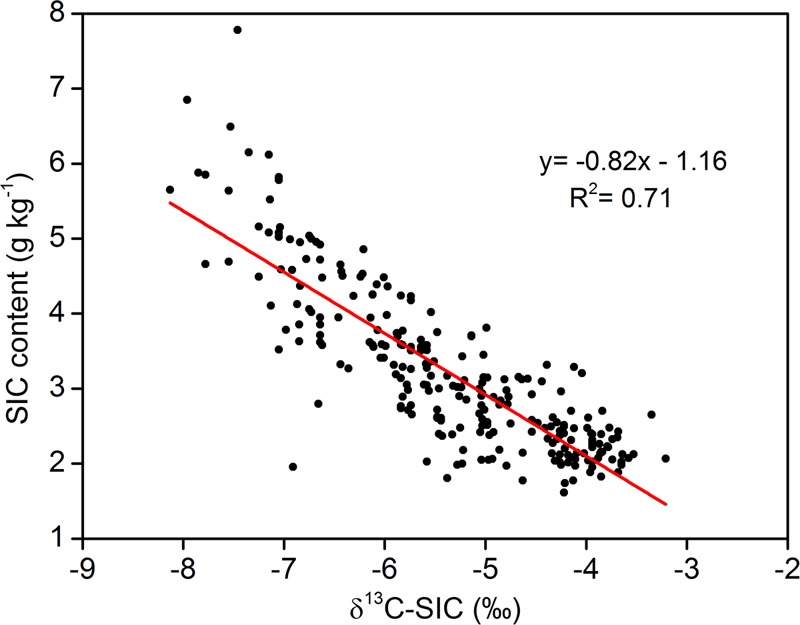
Relationship between soil inorganic δ^13^C value (δ^13^C-SIC) and inorganic carbon (SIC) contents (using all 260 samples within 0–100 cm depth from four sample plots). Significance of the linear regression was considered as *P* < 0.01.

**FIGURE 4:**
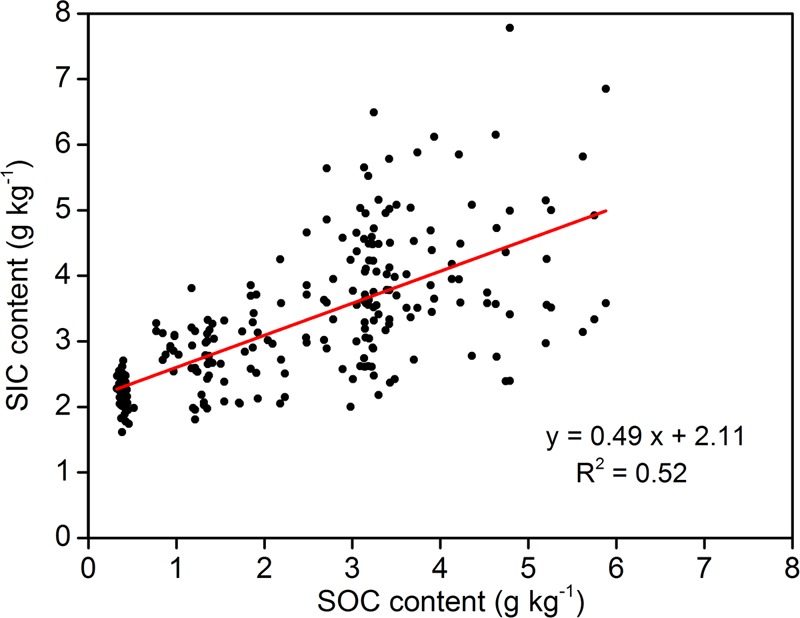
Relationship between soil organic carbon (SOC) and inorganic carbon (SIC) contents (using all 260 samples within 0–100 cm depth from four sample plots). Significance of the linear regression was considered as *P* < 0.01.

**FIGURE 5:**
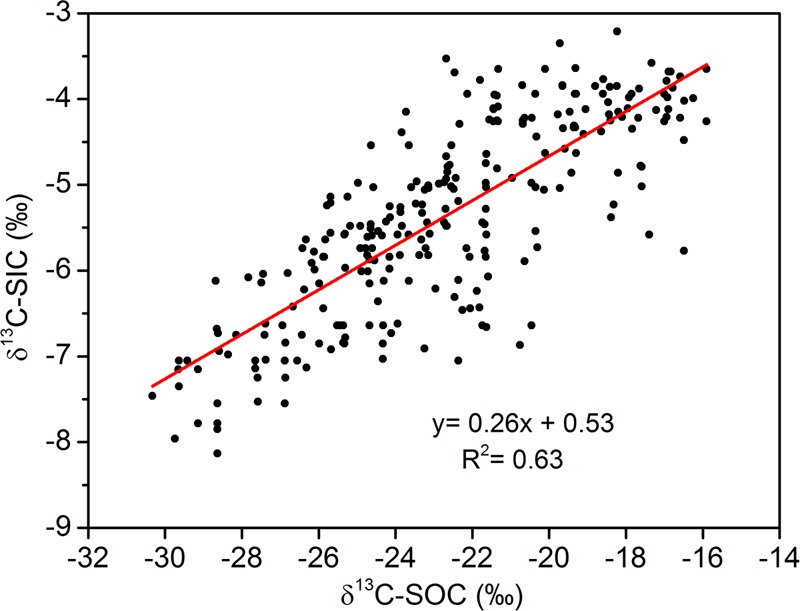
Relationships of soil inorganic δ^13^C value (δ^13^C-SIC) with soil organic δ^13^C value (δ^13^C-SOC) (using all 260 samples within 0–100 cm depth from four sample plots). Significance of the linear regression was considered as *P* < 0.01.

**FIGURE 6:**
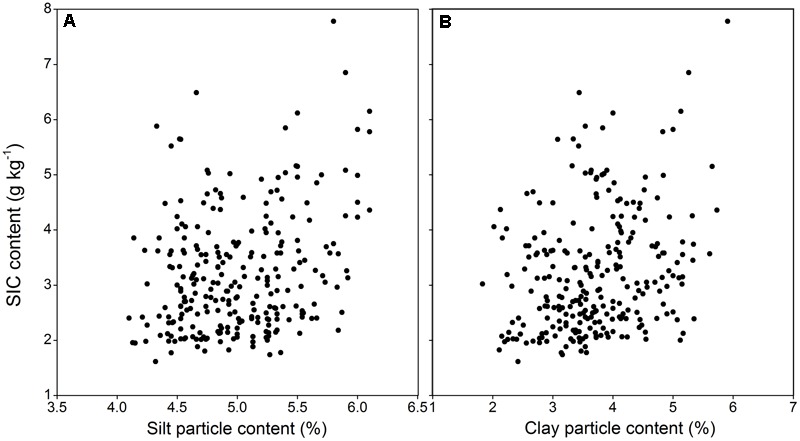
Relationships of soil inorganic δ^13^C value (δ^13^C-SIC) with silt particle content and clay particle content (using all 260 samples within 0–100 cm depth from four sample plots). **(A)** Relationship between δ^13^C-SIC and silt particle content; **(B)** relationship between δ^13^C-SIC and clay particle content. Significance of the linear regression was considered as *P* < 0.01.

**Figure 3** shows a strong correlation between SIC content and δ^13^C-SIC. Using all 260 samples, the relationship between δ^13^C-SIC content and SIC was shown to fit a linear model, and δ^13^C-SIC was observed to explain more than 70% of the variation in SIC (*R*^2^ = 0.71, *P* < 0.01). Our data also showed that the variations in SIC and δ^13^C-SIC were related to SOC and δ^13^C-SOC. There was a positive linear relationship (*R*^2^ = 0.52, *P* < 0.01) between SOC and SIC content for all soil samples (**Figure 4**). The entire dataset (260 samples) exhibited a positive correlation between δ^13^C-SOC and δ^13^C-SIC (*R*^2^ = 0.63, *P* < 0.01, **Figure 5**). Additionally, there was no obvious correlation between silt particle content and SIC content (**Figure 6A**) or between clay particle content and SIC content (**Figure 6B**).

## Discussion

### SIC Sequestration Following Afforestation and the Contribution of Soil Fine Particles to SIC Sequestration

Our results showed that the SIC stock at depth of 0–100 cm in SL was 34.2 Mg ha^-1^ and that it gradually increased along the chronosequence of afforestation (**Figure 2**). The results were consistent with those reported by Su et al. (2010) and Li Y.Q. et al. (2013), who also observed that SIC increased markedly with plantation age after afforestation on SL. However, our findings were in disagreement with some earlier reports in semiarid regions. In the Columbia Plateau, Oregon, United States, after 10 years, poplar plantations in a desert reduced the SIC concentration from 2.6 to 1.2 g kg^-1^ in the surface layer (Sartori et al., 2007). In the Chinese Loess Plateau, Wang et al. (2016) reported that the SIC storage at depth of 0–100 cm in the farmland was significantly lower than that in the restored artificial forestland, with a difference of 16.8 Mg ha^-1^. The SIC reduction in these inconsistent findings was mainly caused by irrigation or surface runoff, which can remove mass containing dissolved inorganic carbon. In the present study, similar processes would not be applicable because there was no irrigation or heavy rainfall. Therefore, our findings indicate that afforestation on shifting SL has a high potential to sequester SIC in degraded semiarid regions.

One theory posits that soil fine particles may play an important role in SIC sequestration following afforestation (Li et al., 2012). Plant canopies can intercept and deposit fine particles from the wind-sand flow after afforestation. This sediment contains rich carbonate sources, such as calcite, and causes a rapid SIC accumulation in surface soil (0–20 cm) (Wang et al., 2006). However, we found that this theory could not provide a complete explanation for SIC accumulation. Afforestation on SL not only elevates SIC stock in the surface soil layer, but also increases SIC levels in the deeper layers (**Table 4**; Li Y.Q. et al., 2013). Nevertheless, afforestation enhanced fine particles only at a depth of 0–40 cm, but not in the 40–100 cm depths (**Table 3**). In the deep layers (>40 cm), soil fine particles stack at an exceptionally slow rate and contribute little to SIC sequestration (Li et al., 2007). Moreover, we detected no correlation between fine particles and SIC content in the present study (**Figure 6**), further suggesting that the contribution of soil fine particles by the canopy to SIC sequestration is limited for the 0–100 cm soil layer. This phenomenon indicates that SIC sequestration is not exclusively derived from fine particle deposition and that other SIC accumulation processes may be occurring after afforestation.

### Effects of Afforestation on Stable Carbon Isotopes and Implications for Revealing the Mechanism of SIC Sequestration

We found that δ^13^C-SIC decreased with plantation age in forestlands (**Table 5**). Wang J.P. et al. (2015) found that the δ^13^C-SIC for desert soil was significantly higher than that for shrubland soil on the northeastern edge of the Taklamakan Desert, China. Liu et al. (2014) also pointed out that the δ^13^C value of soil carbonate along a chronosequence decreased gradually with vegetation restoration. SIC is composed of the LIC and PIC, which have distinct δ^13^C-SIC values. The changes in δ^13^C-SIC following vegetation rehabilitation can be used to explain the reason for SIC variation (Stevenson et al., 2005). There is sufficient evidence that the decrease in δ^13^C-SIC indicates PIC formation when land use patterns change (Jin et al., 2014; Liu et al., 2014; Wang et al., 2014, 2016; Wang J.P. et al., 2015; Bughio et al., 2016). Accordingly, the decrease in δ^13^C-SIC with plantation age in our study indicates that afforestation induced abundant PIC formation. Furthermore, a strong negative linear relationship between δ^13^C-SIC and SIC content in our study (**Figure 3**), which was also observed by Wang X.J. et al. (2015) in the northwest China, suggests that a decreasing δ^13^C-SIC is associated with SIC sequestration following afforestation. Specifically, PIC formation is accompanied by SIC sequestration, as the decrease in δ^13^C-SIC is indicative of the formation of PIC. Therefore, the carbon isotope data in this study indicate that SIC sequestration is probably caused by PIC formation after afforestation on SL. Additionally, an estimation of the amount of PIC would be very important to better understanding the contribution of PIC to SIC sequestration. Based on the precise δ^13^C-SIC, δ^13^C-PIC and δ^13^C-LIC values and empirical formulas, Wang et al. (2014) successfully estimated the accumulation rate of PIC under fertilization for loess soil. This method can ostensibly be used to calculate the amount of PIC in the forestlands in our study. However, an accurate δ^13^C-LIC value in the desert cannot be measured with the current technology, so we cannot supply values for the PIC stocks in this study. The δ^13^C-LIC of desert soil should be precisely identified in future studies because it is crucial for quantifying PIC stock.

### Effect of SOC Accumulation on PIC Formation

In this study, afforestation simultaneously enhanced SIC and SOC contents (**Figure 2**), and SIC content was positively correlated with SOC content (**Figure 4**). Similar relationships have also been identified in other arid and semiarid regions in China (Zhang N. et al., 2010; Wang X.J. et al., 2015; Guo et al., 2016). These results suggest that the increase of SIC following afforestation may be related to SOC accumulation. Furthermore, our results showed that there was a decrease in both δ^13^C-SIC and δ^13^C-SOC with plantation age. δ^13^C-SIC was strongly positively correlated with δ^13^C-SOC (**Figure 5**), a finding that is consistent with the observations of Landi et al. (2003). In other words, the decrease in δ^13^C-SIC was accompanied by a decrease in δ^13^C-SOC. The decrease in δ^13^C-SIC indicates PIC formation, and SOC accumulation invariably leads to a decrease in δ^13^C-SOC due to plant litter input (Trolier et al., 1996; Jin et al., 2014). These results further imply that the PIC formation following afforestation is correlated with SOC accumulation. Soil organic matter affected PIC formation by regulating soil CO_2_ concentration and the precipitation of carbonate in the alkaline environment (Monger et al., 2015). PIC accumulation involves two main reactions:

(3) $${\text{2CO}}_{\text{2}}{\text{+2H}}_{\text{2}}\text{O}↔{\text{2HCO}}_{\text{3}}^{\text{-}}{\text{+2H}}^{\text{+}}$$

(4) $${\text{Ca}}^{\text{2+}}{\text{+2HCO}}_{\text{3}}^{\text{-}}↔{\text{CaCO}}_{\text{3}}{\text{+H}}_{\text{2}}{\text{O+CO}}_{\text{2}}$$

A mass of CO_2_ is released into the soil following shrub and tree plantation in deserts, mainly due to the decomposition of the increased amount of organic matter (Zhang Z.S. et al., 2013). In general, an increase in soil CO_2_ concentration would lead to the production of HCO_3_^-^. The accumulated HCO_3_^-^ can drive reaction (4) to the right, resulting in the precipitation of carbonate (Wang X.J. et al., 2015; Zamanian et al., 2016). When 2 mole of CO_2_ is consumed, 1 mole of CaCO_3_ is generated. At our study site, the soil has a pH greater than 8 (**Table 3**) and is rich in available Ca^2+^ and Mg^2+^ (**Table 1**). The decomposition of the increased SOC in forestlands would dramatically elevate the soil CO_2_ concentration and facilitate the occurrence of reaction (3). The alkaline environmental conditions could neutralize the H^+^ from reaction (3), which may be the reason for the decline in pH in forestlands (**Table 3**). These conditions also continuously promote the formation of HCO_3_^-^. The newly generated HCO_3_^-^ combined with available cations may cause PIC accumulation following afforestation (Meyer et al., 2014; Monger et al., 2015). In addition to the CO_2_ emitted via decomposition of the increased SOC, soil CO_2_ respired by the roots of poplar trees (autotrophic respiration) would affect the formation of PIC. The effects of autotrophic respiration on PIC formation in plantation lands need to be studied in future. Additionally, a long-term study by observing SIC, SOC, soil carbon isotopes, soil CO_2_ concentration and available cations in the same forestland is required, which could more directly and precisely characterize the mechanisms of SIC variation along a chronosequence of afforestation.

## Conclusion

Our data demonstrate that afforestation on shifting SL has a high potential to sequester SIC in degraded semiarid regions. Afforestation elevated soil fine particles only at 0–40 cm, and there was no correlation between SIC content and soil fine particles, suggesting that the contribution of soil fine particle deposition to SIC accumulation is limited. The decrease in δ^13^C-SIC along a chronosequence of forestland and the relationship between δ^13^C-SIC and SIC content both indicate that SIC sequestration following afforestation is probably caused by PIC formation. The positive correlations between SIC content and SOC content and between δ^13^C-SIC and δ^13^C-SOC imply that the newly formed PIC may be closely related to SOC accumulation. Our findings suggest that SIC plays an important role in the carbon cycle in semiarid areas and that by overlooking SIC, we may substantially underestimate carbon sequestration capacities following vegetation rehabilitation. Our stable carbon isotope data will help to form an understanding of the mechanisms of SIC formation and transformation in arid and semiarid areas.

## Author Contributions

JL designed the experiment; YG, JT, and YP carried out the field work; YG and JL analyzed the data; YG wrote the manuscript; and JL assisted with revising the draft manuscript.

## Conflict of Interest Statement

The authors declare that the research was conducted in the absence of any commercial or financial relationships that could be construed as a potential conflict of interest.
